# Assessment of acute poisoning in children using poisoning severity scores: A cross-sectional study at Damietta General Hospital Egypt

**DOI:** 10.1016/j.toxrep.2024.101735

**Published:** 2024-09-13

**Authors:** Heba Youssef Sayed, Rana Elawady, Mona Ibrahim Elyamany, Mohamed S. Hemeda

**Affiliations:** a Department of Forensic Medicine and Clinical Toxicology. Faculty of Medicine – Port Said University, Egypt; b Department of Forensic Medicine and Clinical Toxicology. Faculty of Medicine – Damietta University, Egypt

**Keywords:** Children, Household cleaning products, Pharmaceutical drugs, Petroleum products, Accidental poisoning, Emergency department, Clinical outcomes, Morbidity and mortality, Rural healthcare

## Abstract

Acute poisoning is a prevalent health issue, particularly among children, due to their natural curiosity and tendency to explore. This cross-sectional study aimed to evaluate the characteristics, causes, clinical presentation, and outcomes of acute intoxication in children at Damietta General Hospital, Egypt. We included 106 pediatric patients (aged under 18 years) with a clear history or clinical signs of acute poisoning. Data collection encompassed sociodemographic information, type of poison, mode of poisoning, and clinical outcomes. Poison Severity Score (PSS) was employed to assess the severity. The most affected age group was preschool children (3–6 years), accounting for 41.5 % of cases, with males representing 52.8 %. Accidental poisoning constituted 83 % of cases, with household cleaning products (34 %) and petroleum products (18.9 %) being the most common toxic agents. Clinical outcomes revealed that 33 % of patients experienced morbidity, with electrolyte imbalances being the most prevalent complication. The overall cure rate was 94.3 %, while the mortality rate was 5.7 %. This study highlights the significance of parental education and preventive measures, especially in rural areas, to reduce the risk of pediatric poisoning. The PSS proved useful in guiding clinical care, supporting its further use in pediatric toxicology settings.

## 1 Introduction

According to the World Health Organization, the period between birth and eighteen years is known as childhood. Five developmental phases are distinguished: newborn (ages 0–4 weeks), infant (ages > 4 weeks–1 year), toddler (ages > 1–3 years), preschooler (ages 3–6 years), school-aged (ages 6–11 years), and adolescent (ages 12–18 years) [1]. A common emergency that poses a significant threat to pediatric health is acute intoxication. The patterns of acute intoxication vary between countries and even within regions. Poisoning can result from a variety of causes and is categorized into two types: unintentional and intentional, each with distinct characteristics. Pediatric intoxication can lead to long-term physical and psychological problems, which place a significant financial burden on society [2].

Intoxication has a significant social and economic impact and is the third most frequent emergency in pediatrics. Toxic exposure and poisoning are among the most common global public health issues affecting children [3]. Pediatric intoxication occurs frequently due to the interaction of toxins with the child, their family, and their environment. Due to rapid industrialization, urban lifestyles, increased drug use, and extensive use of household chemicals, and geotechnical materials in rural areas, children are increasingly exposed to harmful chemicals [4].

In the US, 374 children between the ages of 0 and 18 receive treatment in emergency rooms every day, and two of them die from poisoning. In 2019, there were 37.4 poisoning incidents per 1000 children under six years of age, with 99.2 % of these exposures being unintentional and 43 % involving children under six. [5]

During a 20-year evaluation, a total of 26,716 children between the ages of 0 and 18 were hospitalized in the Pediatric Emergency Unit of a tertiary hospital in North-East Nigeria: A 20-Year Retrospective Review (2000–2019). Of these hospitalizations, seventy-six (76) were due to acute poisoning, accounting for 0.28 % of cumulative admissions. The calculated prevalence rate was 2.8 per 1000 children. The magnitude and prevalence of intoxication in Africa are not well-documented, as in 2016, only ten out of 58 nations (17.2 %) had poison information centers. It was estimated that 16,500 deaths in Eastern Africa were attributed to unintentional intoxication, which accounted for 1128,500 cases [6].

Due to the high volume of patients being admitted to hospitals and emergency rooms, pediatric intoxication is considered a serious issue in Egypt. The actual number of intoxication cases may be higher than expected because many go unreported. There are significant regional variations in the types and underlying causes of pediatric acute poisonings, even within the same country [7].

Furthermore, hospital admissions for poisoned patients have significantly increased due to the rising use of over-the-counter medications. In high-income nations, common dangerous substances include household items, pharmaceuticals, toxic plants, insecticides, and infestations by animals and insects. Hydrocarbons, medications, cleaning detergents, paraffin, and kerosene are common toxicants in low- and middle-income societies [8]. Iatrogenic poisoning can occur in younger pediatric age groups accidentally, while in older pediatric age groups, intentional poisoning is more common [9].

As measured by the Poisoning Severity Score, poisoning can be categorized as follows: (0) none; (1) minor; (2) moderate; (3) severe; or (4) lethal. This system aims to comprehensively analyze the situation, accounting for the most severe clinical manifestations [10].

When assessing pediatric patients, understanding common poisonings, as well as the patient's medical history and the poison's effects on the body, is essential. To treat poisoning cases, decontamination, enhanced elimination, antidotes, and supportive care are commonly used [11].

The emergency physician can assess the possible severity of intoxication in a child with known exposure through a thorough clinical evaluation, often supplemented by test data and ECG interpretation. Standardized techniques are widely used to evaluate the extent of poisoning. The Toxic Exposure Surveillance System (TESS) and the Matthew-Lawson scale for barbiturate intoxication are two examples of such methods [12].

The European Community (EC), the International Program on Chemical Safety (IPCS), and the European Association of Poisons Centres and Clinical Toxicologists (EAPCCT) developed the Poisoning Severity Score (PSS). This grading scheme qualitatively evaluates the morbidity caused by different categories of poisoning [13].

Regardless of the type of dangerous material, all patients' poisoning severity was graded using the Poisoning Severity Score (PSS), a grading system. The severity grade in the PSS system is determined by the most severe symptoms or signs based on the presence of specific clinical indications. However, the PSS is not specific to any particular poisoning and applies to various types. Additionally, the PSS method requires the assessment of multiple symptoms and signs [14].

Examples of signs and symptoms are provided for each grade to rate severity. Severity grading should not predict risks or hazards based on factors such as the quantity taken or serum/plasma concentrations; it should solely consider the documented clinical symptoms and signs [11].

There are many substances that children may ingest as poisons, including paracetamol, aspirin, non-steroidal anti-inflammatory drugs, organophosphate chemicals, and corrosive substances [15].

Regular surveillance is necessary to identify trends in specific agents and other factors associated with pediatric intoxication. This helps in developing preventive measures and enables emergency department physicians to diagnose and treat poisoning accurately, based on age and timing [16]. Therefore, this study aims to assess the characteristics, etiology, clinical presentation, and consequences of acute intoxication among pediatric age groups.

## 2 Patients and methods

### 2.1 Ethical approval

The Faculty of Medicine, Port Said University's ethical research committee accepted the entire research plan. (ERN number: (106) FCT814a_002).

### 2.2 Study population

The study included 106 pediatric patients of under 18 years of age, of both genders, who presented between July 1, 2023, and June 30, 2024, to the pediatric department of Damietta General Hospital, Damietta, Egypt, with a clear history of acute poisoning or signs and symptoms suggestive of acute poisoning.

### 2.3 Exclusion criteria

Patients above the age of 18, those with chronic illness or medication history, and patients with allergic drug reactions or unknown causes of long-standing disease were excluded.

This decision was made for several reasons:

1. **Diagnostic complexity**: Differentiating between symptoms of acute poisoning and the effects of chronic diseases or drug interactions in these patients can be challenging and might affect the accuracy of the study results.
2. **Focus on acute poisoning**: The goal of the study was to assess acute poisoning cases without the confounding effects of chronic diseases or pre-existing conditions, providing a clearer picture of the nature of acute poisoning in otherwise healthy children.
3. **Bias prevention**: Including patients with complex medical histories might introduce bias and skew the results toward specific types of poisonings or outcomes, thus detracting from the study's primary objective of assessing common cases of acute poisoning.

### 2.4 Patients consent

Before the participants were included in the study, all of their legal guardians gave their written informed consent, outlining the purpose of the study and the protocols that would be followed.

### 2.5 Methods

All pediatrics incorporated in this study were subjected to the following:

### 2.6 Full history taking

Sociodemographic information (age, gender), socioeconomic level, and the mother's employment status were collected from all cases. The type of substance (medication, chemical, or natural toxin), the number of agents, approximate dosage, form (pill, capsule, liquid, or cream), exposure route, arrival time to the ED (in hours), main complaints, and symptoms at initial admission were documented for all cases.

### 2.7 General examination

Vital signs included temperature, blood pressure, respiration rate, heart rate, and oxygen saturation. The neurological state was assessed using the Glasgow Coma Scale (GCS). Other systems were reviewed, including cardiovascular (CVS) manifestations, gastrointestinal (GIT) manifestations, respiratory manifestations, genitourinary manifestations, skin manifestations, and mouth odor.

Laboratory investigations that include:

a) Routine investigations such as (Serum electrolytes (Na, K), random blood glucose, liver function testing, and renal function tests).

b) Emergency investigations included complete blood count (CBC), electrocardiogram (ECG), arterial blood gas (ABG), and coagulation profile.
c) Toxicological screening test: Urine analysis (rapid drug abuse detection kit in the urine).
d) investigations included choline esterase levels for organophosphorus toxicity and specific drug levels, such as iron.
e) Follow-up investigations.
f) Instrumental investigations included computed tomography (CT), magnetic resonance imaging (MRI), X-ray, and electrocardiogram (ECG).

### 2.8 Clinical staging and severity of poisoning

The Poison Severity Score (PSS) was used to categorize patients into: -Grade 0 (None): asymptomatic, showing no toxicity or symptoms. -Grade I (minor): mild poisoning signs and symptoms. -Grade II (moderate): noticeable poisoning symptoms or signs. -Grade III (severe): symptoms or signs of potentially fatal poisoning. -Grade IV: Death due to fatal poisoning.

### 2.9 Follow-up data

- Management data included hospital stay duration and clinical care (antidotes, GIT decontamination, symptomatic treatment, or observation).
- Patients' outcome data: Data on care outcomes included discharge, admission, referral, and lost follow-up.
- Discharge: cases discharged following full recovery and observation for 6 – 8 hours. Admission: Patients admitted to the pediatric section receive ongoing care and observation until full recovery.
- Referral: Poison control centers receive referrals for cases.
- Lost follow-up Patients who left the hospital without a diagnosis or a full course of treatment.
- **Statistical analysis of data**

The Statistical Package for Social Sciences (SPSS 27.0, IBM/SPSS Inc., Chicago, IL) was used for statistical analysis of the results. Two types of statistical analysis were conducted. Descriptive statistics: Estimates were made for the mean (X), standard deviation (SD), median (Med), and range for skewed data when summarizing continuous data. Qualitative data were presented using frequency and percentage (%). Inferential or analytical statistics

**Inferential or analytical statistics**: Chi-square analysis was used for comparisons between two or more groups. When more than 25 % of cells had a count of fewer than five in tables (>2*2), the Monte Carlo test was used as a correction for the Chi-Square test. When more than 25 % of cells in 2*2 tables had a count of fewer than five, the Fisher Exact test was used as a correction for the Chi-Square test.

**Independent samples t-test (t-test):** Continuous data were used to test for significant differences between two normally distributed groups. The homogeneity of variances and the assumption of normality in each group were confirmed using the Shapiro-Wilk and Levine's tests, respectively.

**Level of significance (P-value (**: The significance level at which the null hypothesis (the hypothesis of no difference) was rejected, as indicated by the P-values associated with the test statistics in all applied tests. This level was set at 0.05, meaning that P-values ≥ 0.05 were considered statistically non-significant, P-values < 0.05 were considered significant, and P-values < 0.01 were considered highly significant.

## 3 Results

A total of 106 cases were recorded in the pediatric department of Damietta General Hospital with a history of severe poisoning. According to the population data in the study (Table 1), the median age was 5.07 ± 4.74 years. Most cases (41.5 %) were in the 3–6 age group, and 52.8 % of cases were male. The data showed that 55.7 % of the cases were from rural areas, and the socioeconomic level of most cases was average (52.8 %), while 59.4 % of the mothers were unemployed.

**Table 1:** Sociodemographic data in the study's cases (n= 106).

**Variables**		**Study cases N = 106**	
**Age (years)**	Mean ± SD	5.07 ± 4.74	
	Median (Range)	Five years (0.75 or 9 months – 17 years)	
		**Number**	**Percent %**
**Age groups**			
**Infants (< 1 year)**		2	1.9
**Toddlers (1: <3 years old)**		22	20.8
**Preschool age group (3: <6 years old)**		44	41.5
**School age group (6: <12 years old)**		21	19.8
**Adolescents (12: 18 years old)**		17	16
**Sex**			
**Male**		56	52.8
**Female**		50	47.2
**Residence**			
**Urban**		47	44.3
**Rural**		59	55.7
**Socioeconomic level**			
**Low**		24	22.6
**Intermediate**		56	52.8
**High**		26	24.5
**Occupational status of mothers**			
**Not working**		63	59.4
**Working**		43	40.6
**Level of school education**			
**Nursery**		68	64.2
**Primary**		24	22.6
**Preparatory**		5	4.7
**Secondary**		9	8.5

Continuous data expressed as mean±SD and median (range)

Categorical data expressed as Number (%)

According to the data on poisoning in the cases studied (Table 2), the pattern of poisoning in most cases was accidental (83.0 %). The most common substances involved were household cleaning products (34.0 %), petroleum products (18.9 %), and medicines (16.0 %). Exposure to toxins by ingestion occurred in 77.4 % of cases. According to the Glasgow Coma Scale (GCS), 81 participants had a GCS of 15, 14 had a GCS between 13 and 14, 7 had a GCS between 9 and 12, and 4 had a GCS ≤ 8 upon arrival at the hospital. Most cases (67 %) had minor poison severity (Grade 1).

**Table 2:** Poison-related data in the cases of the study, mode, type, severity of poisoning, and delay time since exposure. (n= 106).

**Variables**	**Study cases N = 106**	
	**Number**	**Percent%**
**Mode of poisoning**		
**Accidental**	88	83.0
**Suicidal**	18	17.0
**Type of poisons**		
**Household cleaning products**	36	34.0
**Petroleum products**	20	18.9
**Pesticides**	7	6.6
**Drugs (pharmaceuticals)**	17	16.0
**Drugs (abuse)**	8	7.5
**Gas**	8	7.5
**Animal bites**	6	5.7
**Undetermined/unknown**	4	3.8
**Route of poisoning**		
**Oral**	82	77.4
**Bite/sting**	6	5.7
**Inhalation**	8	7.5
**Eye exposure**	4	3.8
**Dermal exposure**	7	6.6
**Delay time since exposure (Hours)**		
**Mean ± SD**	3.41 ± 2.62	
**Median (Range)**	3 (0.5 – 13)	
**GCS**		
**Mean ± SD**	14.17 ± 2.07	
**Median (Range)**	15 (3 – 15)	
**GCS**		
**Fully conscious (GCS = 15)**	81	76.4
**Mild affection (GCS 13 – 14)**	14	13.2
**Moderate affection (GCS 9 – 12)**	7	6.6
**GCS**		
**Severe affection (GCS ≤ 8)**	4	3.8
**Poison severity**		
**Asymptomatic**	12	11.3
**Minor (grade 1)**	71	67.0
**Moderate (grade 2)**	13	12.3
**Severe (Grade 3)**	10	9.4

Continuous data expressed as mean±SD and median (range)

Categorical data expressed as Number (%)

The results of the clinical study (Table 3) showed that 37 cases were hospitalized, of which 20 were admitted to the Pediatric Intensive Care Unit (PICU), while the remaining patients were transferred to the ward. Regarding final clinical outcomes, the morbidity rate was 33 %. Electrolyte disorders were the most common, affecting 31.4 % of cases, followed by respiratory failure (28.6 %), coma (22.9 %), and convulsions (14.3 %). Other complications included upper gastrointestinal bleeding (11.4 %), aspiration pneumonia (8.6 %), hospital-acquired infections, arrhythmias (5.7 %), acute kidney injury, transient hypertension, and acute liver injury (each 2.9 %). The recovery rate was 94.3 %, while the mortality rate was 5.7 %.

**Table 3:** Clinical outcomes in the cases of the study (n=106).

**Variables**	**Study cases N = 106**	
	**Number**	**Percent%**
**Disposition**		
**Admitted**	37	34.9
**Discharged**	69	65.1
**Site of admission**		
**Ward**	17	45.9
**PICU**	20	54.1
**Final clinical outcome**		
**Morbidity**	35	33
• **Electrolyte disturbances**	11	31.4
• **Respiratory failure**	10	28.6
• **Coma**	8	22.9
• **Convulsions**	5	14.3
• **Upper GIT injury**	5	14.3
• **Aspiration pneumonia**	4	11.4
• **Hospital-acquired infection**	**3**	**8.6**
• **Dysrhythmia**	**2**	**5.7**
• **Acute kidney injury**	**2**	**5.7**
• **Transient hypertension**	**1**	**2.9**
• **Acute liver injury**	**1**	**2.9**
**Cure**	**100**	**94.3**
**Mortality**	**6**	**5.7**

Regarding the comparison of poisoning data by age groups (Table 4), the results showed a statistically significant difference between age groups in terms of the pattern of poisoning (p< 0.001). All children under one year of age (n=2), young children (1–<3 years) (n=22), and preschoolers (3–<6 years) (n=44) were accidentally poisoned. Most participants between the ages of 12 and <18 (n=17) were poisoned as a result of suicide attempts (88.7 %).

**Table 4:** Monte Carlo test comparing mode, types, and routes of poisoning data with age groups of acutely intoxicated pediatric patients.

**Variables**	**Infants (<1 year)** [N=2]	**Toddlers (1:<3 years)** [N=22]	**Preschool age group (3:<6 years)** [N=44]	**School age group (6: <12 years)** [N=21]	**Adolescents (12:18 years)** [N=17]	**Test of Sign.**	**P value**
**Mode of poisoning**							
Accidental	2 (100 %)	22 (100 %)	44 (100 %)	18 (85.7 %)	2 (11.8 %)	MC = 75.742	< 0.001*
Suicidal	0 (0 %)	0 (0 %)	0 (0 %)	6 (14.3 %)	15 (88.2 %)		
**Type of poisons**							
Household cleaning products	1 (50 %)	8 (36.4 %)	21 (47.7 %)	5 (23.8 %)	1 (5.9 %)	MC = 45.464	< 0.001*
Petroleum products	0 (0 %)	5 (22.7 %)		4 (19 %)	1 (5.9 %)		0.077
Pesticides	0 (0 %)	2 (9.1 %)	2 (4.5 %)	1 (4.8 %)	2 (11.8 %)		0.248
Drugs (pharmaceuticals)	1 (50 %)	1 (4.5 %)	3 (6.8 %)	3 (14.3 %)	9 (52.9 %)		< 0.001*
Drugs (abuse)	0 (0 %)	2 (9.1 %)	4 (9.1 %)	0 (0 %)	2 (11.8 %)		0.145
Gas	0 (0 %)	1 (4.5 %)	2 (4.5 %)	4 (19 %)	1 (5.9 %)		0.106
Animal bites	0 (0 %)	1 (4.5 %)	2 (4.5 %)	3 (14.3 %)	0 (0 %)		0.090
Undetermined/unknown	0 (0 %)	2 (9.1 %)	0 (0 %)	1 (4.8 %)	1 (5.9 %)		0.172
**Rout of poisoning**							
Oral	2 (100 %)	15 (68.2 %)	35 (79.5 %)	13 (61.9 %)	16 (94.1 %)	MC= 24.602	0.010*
Bite/sting	0 (0 %)	1 (4.5 %)	2 (4.5 %)	3 (14.3 %)	0 (0 %)		0.110
Inhalation	0 (0 %)	1 (4.5 %)	2 (4.5 %)	4 (19 %)	1 (5.9 %)		0.066
Eye exposure	0 (0 %)	0 (0 %)	3 (6.8 %)	1 (4.8 %)	0 (0 %)	MC= 24.602	0.542
Dermal exposure	0 (0 %)	5 (22.7 %)	2 (4.5 %)	0 (0 %)	0 (0 %)		0.040*

MC: Monte Carlo test

*: Statistically significant (p< 0.05)

Differences between age groups were statistically significant about household cleaning products and medicines (p < 0.001). The largest proportion of exposure to household cleaning products was in the infant, toddler, and preschool age groups (36.4 %, 47.7 %, and 23.8 %, respectively), compared to other age groups. In contrast, the highest percentage of drug exposure was among adolescents (12–<18 years) (52.9 %).

Infant and young age groups were not at risk of unspecific/unknown poisoning, while adolescents were at the highest risk. There was a statistically significant difference between age groups in the modes of exposure to oral and skin poisoning (p=0.010 and 0.040, respectively). Oral exposure was the most common in all age groups: infants, toddlers, preschoolers, school-aged children, and adolescents (100 %, 68.2 %, 79.5 %, 61.9 %, and 94.1 %, respectively). Cutaneous exposure was 22.7 % and 4.5 % in toddlers and preschoolers, respectively.

Table 5 shows a comparison of the degree of severity of intoxication between different age groups. There were statistically significant differences in symptoms between age groups, particularly for asymptomatic cases (p=0.046) and severe symptoms (Grade 3) (p=0.048). All infants showed mild symptoms (Grade 1). Only 18.2 % of young children and preschoolers were asymptomatic. The highest percentage in all age groups (infants, toddlers, preschoolers, school-aged children, and adolescents) had mild symptoms (Grade 1) at 100 %, 63.6 %, 63.6 %, 81.0 %, and 58.8 %, respectively. The largest proportion of severe symptoms (Grade 3) was among adolescents (23.5 %).

**Table 5:** Monte Carlo test Comparing poisoning severity with age groups of acutely intoxicated pediatric patients.

**Variables**	**Infants (<1 year)** [N=2]	**Toddlers (1: <3 years)** [N=22]	**Preschool age group (3: <6 years)** [N=44]	**School age group (6: <12 years)** [N=21]	**Adolescents (12:18 years)** [N=17]	**Test of Sign.**	**P value**
**Poison severity**							
Asymptomatic	0 (0 %)	4 (18.2 %)	8 (18.2 %)	0 (0 %)	0 (0 %)	MC =13.973	0.040*
Minor (grade 1)	2 (100 %)	14 (63.6 %)	28 (63.6 %)	17 (81 %)	10 (58.8 %)		0.070
Moderate (grade 2)	0 (0 %)	3 (13.6 %)	5 (11.4 %)	2 (9.5 %)	3 (17.6 %)		0.126
**Poison severity**							
Severe (Grade 3)	0 (0 %)	1 (4.5 %)	3 (6.8 %)	2 (9.5 %)	4 (23.5 %)		0.034*

MC: Monte Carlo test

*: Statistically significant (p< 0.05)

The differences in mode and severity of poisoning were statistically significant (Table 6). All asymptomatic patients (Grade 0) and 88.7 % of patients presenting with minor symptoms (Grade 1) were exposed to poison accidentally.

**Table 6:** One-way analysis ANOVA test for comparing mode, type, and route of poisoning with poisoning severity score of acutely intoxicated pediatric patients.

	**Asymptomatic** **(grade 0)**	**Minor (grade 1)**		**Moderate (grade 2)**	**Severe (grade 3)**	**Test of Sign.**	**P value**
**Mode of poisoning**	[N=12]	[N=71]		[N=13]	[N=10]		
Accidental	12 (100 %)	63 (88.7 %)		8 (61.5 %)	5 (50 %)	MC= 16.087	0.001*
Suicidal	0 (0 %)	8 (11.3 %)		5 (38.5 %)	5 (50 %)		
**Type of poisons**							
Household cleaning products	11 (91.7 %)	25 (35.2 %)		0 (0 %)	0 (0 %)	MC = 63.978	< 0.001*
Petroleum products	0 (0 %)	18 (25.4 %)		1 (7.7 %)	1 (10 %)		0.042*
Pesticides	0 (0 %)	4 (5.6 %)		3 (23.1 %)	0 (0 %)		0.048*
Drugs (pharmaceuticals)	0 (0 %)	8 (11.3 %)		7 (53.8 %)	2 (20 %)		< 0.001*
Drugs (abuse)	1 (8.3 %)	3 (4.2 %)		1 (7.7 %)	3 (30 %)		0.058
Toxic gases	0 (0 %)	5 (7 %)		1 (7.7 %)	2 (20 %)		0.066
Animal bites	0 (0 %)	4 (5.6 %)		0 (0 %)	3 (30 %)		0.042*
Undetermined/ Unknown	0 (0 %)	4 (5.6 %)		0 (0 %)	0 (0 %)		0.316
**Rout of poisoning**							
Oral	8 (66.7 %)		56 (78.9 %)	12 (92.3 %)	6 (60 %)	*MC = 19.534*	0.054
Bite/sting	0 (0 %)		4 (5.6 %)	0 (0 %)	2 (20 %)		0.072
Inhalation	0 (0 %)		5 (7 %)	1 (7.7 %)	2 (20 %)		0.074
Eye exposure	1 (8.3 %)		3 (4.2 %)	0 (0 %)	0 (0 %)		0.230
Dermal exposure	3 (25 %)		4 (5.6 %)	0 (0 %)	0 (0 %)		0.044*

F= One-way ANOVA MC: Monte Carlo test

*: Statistically significant (p< 0.05)

Regarding the relationship between the type of poison and the severity of poisoning, there were statistically significant differences between household cleaning products, petroleum products, pesticides, pharmaceutical drugs, animal bites, and severity of poisoning (p= 0.001, <0.001, 0.042, 0.048, <0.001, 0.042, respectively). Most patients with asymptomatic presentation (91.7 %) were exposed to household cleaning products, while 35.2 % of patients with minor symptoms were exposed to household cleaning products. The majority of patients (53.8 %) had moderate symptoms (Grade 2) after being exposed to pharmaceutical drugs.

There was no statistically significant difference between the severity of poisoning and other routes of exposure. However, there was a statistically significant difference between dermal exposure and the severity of poisoning (p= 0.044).

Clinical outcomes were linked to the severity of intoxication, as shown in Table 7. All asymptomatic patients were discharged from the hospital and survived. Among Grade 1 patients, 80.3 % were discharged, while the rest were admitted, with 78.6 % of those admitted being placed in the ward. All Grade 1 patients survived. Grade 2 and 3 patients were all admitted to the hospital, with 61.5 % of Grade 2 patients and 90.0 % of Grade 3 patients admitted to the ICU. The survival rates of patients in Grades 2 and 3 were 84.6 % and 60 %, respectively.

**Table 7:** Monte Carlo test Comparing the clinical outcomes with the poison severity score of acutely intoxicated pediatric patients.

	**Asymptomatic (grade 0)**	**Minor (grade 1)**	**Moderate (grade 2)**	**Severe (grade 3)**	**Test of Sign.**	**P value**
Disposition	[N=12]	[N=71]	[N=13]	[N=10]		
Discharged	12 (100 %)	57 (80.3 %)	0 (0 %)	0 (0 %)	MC= 56.534	< 0.001*
Admitted	0 (0 %)	14 (19.4 %)	13 (100 %)	10 (100 %)		
Site of admission (n= 37)	[N=0]	[N=14]	[N=13]	[N=10]		
Ward	–	11 (78.6 %)	5 (38.5 %)	1 (10 %)	MC= 11.496	0.003*
ICU	–	3 (21.4 %)	8 (61.5 %)	9 (90 %)		
Outcome	[N=12]	[N=71]	[N=13]	[N=10]		
Cure rate	12 (100 %)	71 (100 %)	11 (84.6 %)	6 (60 %)	MC= 29.365	< 0.001*
Mortality rate	0 (0 %)	0 (0 %)	2 (15.4 %)	4 (40 %)		

MC: Monte Carlo test

*: Statistically significant (p< 0.05)

The relationship between morbidity and age groups, pattern of poisoning, type of poisoning, and mode of exposure to poisoning is illustrated in Table 8. There were 71 cases without complications and 35 with complications. Regarding the relationship between morbidity and age groups, the highest percentage of cases without complications and those with complications was among infants and preschoolers (3–6 years), at 42.3 % and 40 %, respectively. Of the 62 patients (87.3 %) who did not experience complications, poisoning was accidental, while 26 patients (74.3 %) who had complications were also accidentally poisoned.

**Table 8:** Chi-square test and Monte Carlo test showing the relation between morbidity rate and age groups, mode of poisoning, type of poisoning, and route of poisoning of acutely intoxicated pediatric patients**.**

**Variables**	**No morbidities [N= 71]**	**Morbidities [N= 35]**	**Test of Sign.**	**P value**
**Age groups**				
**Infants (< 1 year)**	**2 (2.8 %)**	**0 (0 %)**	**MC= 2.099**	**0.088**
**Toddlers (1: <3 years old)**	**16 (22.5 %)**	**6 (17.1 %)**		**0.218**
**Preschool age group (3: <6 years old)**	**30 (42.3 %)**	**14 (40 %)**		**0.340**
**School age group (6: <12 years old)**	**13 (18.3 %)**	**8 (22.9 %)**		**0.567**
**Adolescents (12: 18 years old)**	**10 (14 %)**	**7 (20 %)**		**0.592**
**Mode of poisoning**				
**Accidental**	**62 (87.3 %)**	**26 (74.3 %)**	**χ2= 2.827**	**0.093**
**Suicidal**	**9 (12.7 %)**	**9 (25.7 %)**		
**Type of poisons**				
Household cleaning products	34 (47.9 %)	2 (5.7 %)	MC = 33.105	< 0.001*
Petroleum products	14 (19.7 %)	6 (17.1 %)		0.836
Pesticides	3 (4.2 %)	4 (11.4 %)		0.272
Drugs (pharmaceuticals)	8 (11.3 %)	9 (25.7 %)		0.039*
Drugs (abuse)	1 (1.4 %)	7 (20 %)		0.002*
Gas	3 (4.2 %)	5 (14.3 %)		0.106
Animal bites	4 (5.6 %)	2 (5.7 %)		0.946
Undetermined/ Unknown	4 (5.6 %)	0 (0 %)		0.648
**Rout of poisoning**				
Oral	8 (66.7 %)	26 (74.3 %)	MC = 6.337	0.074
Bite/sting	3 (4.2 %)	3 (8.6 %)		0.552
Inhalation	3 (4.2 %)	5 (14.3 %)		0.094
Eye exposure	4 (5.6 %)	0 (0 %)		0.342
Dermal exposure	6 (8.5 %)	1 (2.9 %)		0.310

χ2= Chi-square test MC: Montecarlo test

*: Statistically significant (p< 0.05).

The highest percentage of patients who did not experience complications were exposed to household cleaning products (47.9 %), while the lowest percentage was exposed to drugs (1.4 %). The highest percentage of patients who experienced complications were exposed to medications (25.7 %). Patients without complications and those with complications experienced oral poisoning at rates of 66.7 % and 74.3 %, respectively. There was no statistically significant difference between the two groups in relation to the method of poisoning (oral, bite/sting, inhalation, eye exposure, and skin exposure).

Table 9 shows the relationship between mortality, age groups, pattern of poisoning, type of poisoning, and mode of exposure to poisoning. Of the 100 patients who survived, the largest proportion were preschoolers (42.0 %), while the lowest percentage of survivors were infants (2.0 %). Additionally, six patients died, with the largest proportion being preschoolers (33.3 %), and there were no deaths among infants**.**

**Table 9:** Fischer’s exact test and Monte Carlo test showing the relation between mortality rate and age groups, mode of poisoning, type of poisoning, and route of poisoning of acutely intoxicated pediatric patients**.**

**Variables**	**Survived** [N= 100]	**Died** [N= 6]	**Test of Sign.**		**P value**
**Age groups**					
Infants (< 1 year)	2 (2.0 %)	0 (0 %)	MC= 6.108		0.866
Toddlers (1: <3 years old)	21 (21.0 %)	1 (16.7 %)			0.752
Preschool age group (3: <6 years old)	42 (42.0 %)	2 (33.3 %)		0.514	
School age group (6: <12 years old)	21 (21.0 %)	0 (22.9 %)		0.964	
Adolescents (12: 18 years old)	14 (14.0 %)	3 (50 %)		0.062	
**Mode of poisoning**					
Accidental	85 (85.0 %)	3 (50 %)	FET= 4.919	0.027*	
Suicidal	15 (15.0 %)	3 (50 %)			
**Type of poisons**					
Household cleaning products	36 (36.0 %)	0 (0 %)	MC = 11.765	0.002*	
Petroleum products	18 (18.0 %)	2 (33.3 %)		0.473	
Pesticides	6 (6.0 %)	1 (16.7 %)		0.630	
Drugs (pharmaceuticals)	17 (17.0 %)	0 (0 %)		**0.156**	
Drugs (abuse)	6 (6.0 %)	2 (33.3 %)		0.028*	
Gas	7 (7.0 %)	1 (16.7 %)		0.676	
Animal bites	6 (6.0 %)	0 (0 %)		0.708	
Undetermined/ Unknown	4 (4.0 %)	0 (0 %)		0.824	
**Rout of poisoning**					
Oral	76 (76.0 %)	5 (83.3 %)	MC = 1.690		0.608
Bite/sting	6 (6.0 %)	0 (0 %)			0.743
Inhalation	7 (7.0 %)	1 (16.7 %)			0.668
Eye exposure	4 (4.0 %)	0 (0 %)			0.816
Dermal exposure	7 (7.0 %)	0 (0 %)			0.688

FET= Fischer’s exact test MC: Montecarlo test

*: Statistically significant (p< 0.05)

Regarding the pattern of poisoning, there was a statistically significant difference (p=0.027); 85.0 % of survivors were accidentally poisoned, while 50 % of the deceased were accidentally poisoned. Most survivors were exposed to household cleaning products (36 %), while only 4 % were exposed to unspecified toxins. Deceased patients exposed to petroleum products and drugs were equal (33.3 %), and the percentages of patients exposed to pesticides or gas were also equal (16.7 %). None of the deceased patients were exposed to household cleaning products, medicines, animal bites, or unspecified toxins. 76.0 % of survivors and 83.3 % of the deceased were poisoned via ingestion.

## 4 Discussion

Acute intoxication in pediatrics remains a significant public health problem and is a frequent reason for hospital admissions, with prevalence rates ranging from 0.33 % to 7.6 %. Most cases involve children aged one to five years, representing 80 % of incidents [17]. In one study evaluating acute poisonings in Shenyang, China, between 2012 and 2016, a mortality rate of 3–5 % was reported. However, it is important to note that these findings are specific to a limited geographic area and may not be representative of broader global trends [18].

he Poison Severity Score (PSS) classifies poisoning severity into five levels: none (0), minor (1), moderate (2), severe (3), and fatal (4). Patterns and poisoning risks differ by age and country, necessitating country-specific epidemiological surveillance for effective prevention [19]. More studies are needed to evaluate the utility of the PSS in pediatric poisoning.

This study was conducted to characterize the pattern of acute pediatric poisoning in Egypt and assess the usefulness of the PSS in these patients. To achieve this, it included 106 pediatric patients with acute intoxication who were recruited from Damietta General Hospital over a one-year period, from July 1, 2023, to June 30, 2024.

There were 106 pediatric patients in this study with a median age of five years, ranging from 9 months to 17 years. Regarding age and sociodemographics, most acute intoxication cases were between the ages of 3 and 6 years (41.5 %); the 1–<3-year age group accounted for 20.8 % of cases, followed by the 6–12-year and 12–18-year age groups (19.8 % and 16 %, respectively). The least represented age group was under one year (1.9 %).

This finding highlights that pediatric intoxication is a significant public health problem. Children exhibit exploratory behavior, are curious about their surroundings, are unaware of potential dangers, and frequently have accidents—especially when they put liquids or colorful items in their mouths. In agreement with the current study, Abdelhamid et al., in previous research on Egyptian patients, demonstrated that most intoxication cases occurred in children under seven years of age (26.4 %) [20].

. In concordance with the current study, Kandeel et al., in another study on 760 patients, found that 62.1 % of intoxication cases occurred in the 2–6-year age range [21]. In contrast, Moon et al., in a study involving 6409 children, reported that most patients were either under two years of age (34.2 %) or between the ages of 13 and 18 years (32.6 %), with a statistically significant difference (p< 0.001) [22].

The present study showed a nearly equal sex distribution regarding acute intoxication, with a slight male predominance (52.8 %), likely due to boys being more active than girls and thus more likely to come into contact with toxic substances inadvertently.

In agreement with the present study, Kandeel et al. found that 55 % of intoxication patients were male [21]. In contrast, Moon et al., in a previous study, demonstrated that 52.4 % of patients were female [13].

Regarding residency, the majority of patients in the current study were from rural areas (55.7 %). This may be explained by the lower socioeconomic status and educational levels in rural areas, making household cleaning products and pharmaceuticals more readily accessible to children.

Similarly, Kandeel et al., in a previous study, demonstrated that more poisoning cases occurred in rural areas compared to urban areas [21]. This contrasts with the findings of Alazab, who observed a higher occurrence of poisoning in metropolitan regions (79.9 %) compared to rural areas (20.1 %) in Egypt [23]. This discrepancy could be attributed to the fact that Alazab's study was conducted in an area that is predominantly urban in nature. Most mothers of the included children were not working (59.4 %). On the other hand, Disfani et al. found that higher employment among mothers was associated with increased odds of acute pediatric intoxication by 2.8 times as compared to non-working mothers (p< 0.001) [24]."

The present study showed that most acute intoxication cases occurred at nursery (64.2 %) or primary stages (22.6 %) of education. This is explained by the high curiosity of pediatrics at this age. In a study by Çalışkan et al., it was found that most acute intoxication cases occurred during the nursery stage [26]. Similarly, in the current study, we observed that the majority of intoxication cases also happened in the nursery stage [25].

Oral administration accounted for 77.4 % of instances, while other routes (such as inhalation, cutaneous, IV, etc.) accounted for only 22.6 % of cases. This could be due to toxic substances being stored in easily accessible areas and packaged in visually appealing colors or containers that attract children A similar outcome was found in other research. In this study, most cases of acute intoxication were accidental (83 %), with 17 % of patients attempting suicide. Consistent with the current study, Moon et al. reported that 75.3 % of intoxicated children were accidentally poisoned [22]. In contrast, a previous study conducted in England reported a higher rate of intentional poisoning in children [26]. This difference may reflect the lower incidence of suicide attempts among the included patients and the reduced use of poisons as a method of suicide, possibly due to traditional and religious beliefs. According to the present study, the most reported types of poisons were household cleaning products (34 %), followed by petroleum products (18.9 %), and pharmaceutical drugs (16 %). Drugs of abuse and gas accounted for 7.5 % each, while pesticides made up 6.6 % of cases. Animal bites were detected in 5.7 % of patients. The cause of intoxication was unknown in 3.8 % of cases in this study In agreement with the present results, previous studies have demonstrated that the most frequently involved substances in accidental poisoning vary by region. Household products are most commonly involved in the Eastern Mediterranean and Western Europe [27]. In contrast to this study, therapeutic medications are most frequently used in cases of poisoning in the Americas and the Western Pacific [28]. In a study by Moon et al., the most reported type of poison was pharmaceutical drugs (50.6 %), followed by gas (14.5 %), while household cleaning products accounted for only 10.6 % and petroleum products 4.3 % [22]. These differences may be related to the availability of household cleaning products to young children and the safety precautions, such as child-resistant packaging, used for pharmaceutical medications and chemical products, which vary by region. At At presentation, most patients were fully conscious (76.4 %), while 13.2 % had mild impaired consciousness. Consistent with the current study, Moon et al. reported that 97.8 % of the included patients were referred to the hospital fully conscious [22]. According to the Poison Severity Score, 11.3 % of patients were asymptomatic. Most patients had a minor degree of poisoning (67 %), while 12.3 % had a moderate degree and 9.4 % had a severe degree of poisoning. In agreement with the present study, Abdelhamid's previous study showed that 79.4 % of patients presented with mild symptoms [20].

Regarding the final clinical outcome, 65.1 % of the included patients were discharged from the emergency room, while 34.9 % were admitted. Of those admitted, 45.9 % were placed in the ward, and 54.1 % were admitted to the ICU. Another study by Hassan et al. showed that 86 % of patients were discharged, while 10.3 % were admitted to the ICU [29]. In contrast, Kandeel et al. demonstrated that 74.6 % of patients in his study were admitted for 1–3 days, and only 16 % of patients were discharged on the referral day [21].

In the present study, the morbidity rate was 35 %, and causes of morbidity included electrolyte disturbances (31.4 %), respiratory failure (28.9 %), coma (22.9 %), and convulsions (14.3 %). The most commonly reported morbidities were upper GIT injury (14.3 %), aspiration pneumonia (11.4 %), and hospital-acquired infection (8.6 %).

Other causes of morbidity occurred less frequently. In agreement with the present study, Patel et al., in another study, showed that the most common causes of morbidity were respiratory issues (35.9 %), cardiac problems (33.5 %), and coma or other neurological issues (26.6 %) [30 Similarly, Hassan et al. demonstrated that the gastrointestinal system was the most affected (60 %), followed by the cardiopulmonary system (12.6 %) and the neurological system (10.6 %) [29]. The mortality rate was 5.7 %, and the cure rate was 94.3 %. According to the PSS, this could be explained by the fact that most cases presented with mild or moderate symptoms.

significant variations were observed between the various age groups. Suicidal intoxication was more common among older age groups (p< 0.001). In agreement with the current study, a previous study demonstrated a statistically significant difference between different age groups regarding the mode of poisoning. In older age groups (school-age and adolescence), most of the cases were suicidal (p< 0.001) [23].

The types of poisons differed significantly between different age groups. The most reported poison in younger age groups (less than one year, 1–3 years, and 3–6 years) was household products (p< 0.001). In the 12–18-year age group, the most reported poison was pharmaceutical drugs (p< 0.001).

. In agreement with the present study, a previous study on Egyptian patients showed that most intoxication cases in younger age groups were caused by household cleaning products, while pharmaceutical drugs were more common in older age groups (p< 0.001) [20].

Mintegi et al. discovered similar findings, indicating that the most prevalent toxic substance in the younger age group was household products, and in the older age group was ethanol and drugs [27]. In addition, another study by Kandeel et al. demonstrated that household cleaning products were more frequent in patients aged below six years, while drugs were the commonest poison in children aged above 12 years [21].

The present study showed that mortality and cure rates were comparable between the studied groups. In contrast, Abdelhamid's study did not find significant differences in mortality rates between younger and older children (0.4 % vs. 0.9 %; p>0.05) [20].

On the contrary, Kandeel et al. showed that the majority of fatal cases (86.7 %)belonged to the 12- to 18-year-old age range, with the lowest percentage(13.3 %)occurring in the under-2-year age range. This could be explained by the fact that the smaller amounts consumed by the younger age groups were due to unintentional incidents. Conversely, the increased death rate among older children, who were primarily female, can be explained by the fact that many of these cases involved substantial amounts of intentionally poisoned poisons. [21].

The current study found statistically significant differences in the types of poisons between the various grades of poison severity. Most asymptomatic patients or patients with minor manifestations were intoxicated by household cleaning products (p< 0.001). The most common poisons for moderate disease severity were pesticides (23.1 %) and pharmaceutical drugs (53.8 %). The most common poisons for severe disease were drug abuse (30 %), animal bites (30 %), pharmaceutical drugs, and toxic gases (20 %), in disagreement with the present study. As demonstrated by the current study, all patients with moderate or severe symptoms (grades 2 and 3) were admitted to the hospital, and most of them (61.5 % and 90 %) were admitted to the ICU with statistically significant differences (p= 0.003; < 0.001). In agreement with the current study, Çalışkan et al., in previous research on 444 patients, showed that a higher percentage of patients with moderate and severe degrees of intoxication were admitted to the ICU than patients with minor degrees of intoxication (p< 0.001) [25].

In this study, the cure rate was significantly lower. The mortality rate was higher in patients with moderate or severe manifestations compared to asymptomatic patients or patients with minor manifestations with statistically significant differences (p< 0.001). In concordance with the present study, Abdelhamid, in a previous study, showed that mortality rates were significantly higher in patients presenting with severe manifestations (7.8 %) than mild (0.01 %) or moderate (1.1 %) manifestations (p< 0.001) [20]. Also, another study showed that the mortality rate among severe cases was 86.7 %, while it was 13.3 % in moderate cases and 0 % in minor instances [21].

On the contrary, Çalışkan et al. did not find the presence of significant differences between patients with variable degrees of toxicity as regards cure or mortality rates, as none of the included patients in his study died [25].

## 5 Conclusion

Conclusion: The study showed that acute poisoning in children remains a common cause of emergency admissions. Most poisoning cases occurred accidentally, particularly in younger children under six years old, and were caused by household cleaning products. Suicide attempts involving pharmaceutical drugs were the major cause of poisoning in older children. Morbidity was primarily due to respiratory and neurological complications, while the mortality rate was low. The Poison Severity Score (PSS) proved to be a useful tool for assessing the severity and outcome of poisoning. The results highlight the importance of poison control programs and the need to raise awareness among parents and caregivers, especially in rural areas, to prevent such incidents.

## Ethical statement

This study was conducted in accordance with the ethical standards of the institutional and national research committee and with the 1964 Helsinki Declaration and its later amendments or comparable ethical standards. The study protocol was reviewed and approved by the Ethical Research Committee of the Faculty of Medicine, Port Said University (ERN number: 106 FCT814a_002).

Informed consent was obtained from the legal guardians of all participants included in the study. Participation was voluntary, and the confidentiality of the participants was maintained throughout the research process.

## Sources of Funding

None.

## CRediT authorship contribution statement

**Mohamed hemeda:** Writing – review & editing, Writing – original draft, Visualization, Validation, Supervision, Software, Resources, Project administration, Methodology, Investigation, Funding acquisition, Formal analysis, Data curation, Conceptualization. **Rana Elawady:** Writing – review & editing, Writing – original draft, Visualization, Validation, Supervision, Software, Resources, Project administration, Methodology, Investigation, Funding acquisition, Formal analysis, Data curation, Conceptualization. **Heba Youssef Sayed:** Writing – review & editing, Writing – original draft, Visualization, Validation, Supervision, Software, Resources, Project administration, Methodology, Investigation, Funding acquisition, Formal analysis, Data curation, Conceptualization. **Mona Ibrahim Elyamany:** Writing – review & editing, Writing – original draft, Visualization, Validation, Data curation, Conceptualization.

## Declaration of Competing Interest

The authors declare that they have no known competing financial interests or personal relationships that could have appeared to influence the work reported in this manuscript.

## Data Availability

No data was used for the research described in the article.

## References

[1] De Onis M. (2017). Child growth and development. Nutr. Health a Dev. World.

[2] Soave P.M., Curatola A., Ferretti S., Raitano V., Conti G., Gatto A., Chiaretti A. (2022). Acute poisoning in children admitted to the pediatric emergency department: a five-year retrospective analysis. Acta Bio Med.: Atenei Parm..

[3] Dayasiri M.B.K.C., Jayamanne S.F., Jayasinghe C.Y. (2018). Patterns and outcome of acute poisoning among children in rural Sri Lanka. BMC Pediatr..

[4] Gerald, F. (2020). *Analysis of Children’s Health Risks Due To Exposure To Home Environmental Toxicants in Highly Congested Urban Areas in Dar Es Salaam City* (Doctoral dissertation, Ardhi University).

[5] Gummin D.D., Mowry J.B., Beuhler M.C., Spyker D.A., Bronstein A.C., Rivers L.J., Pham N.P.T., Weber J. (2021 Dec). 2020 Annual Report of the American Association of Poison Control Centers' National Poison Data System (NPDS): 38th Annual Report. Clin. Toxicol. (Philos. ).

[6] Isaac W.E., Iliya J., Adamu S., Apllos D., Oyeniyi C. (2022). The spectrum of poisoning and outcome among children in a tertiary hospital, North-East Nigeria: a 20 years retrospective review, 2000-2019. Open J. Pediatr..

[7] Farag A.A., Said E.A., Fakher H.M. (2020). Patterns of pediatric acute poisoning at banha poisoning control center, Egypt: a one-year prospective study. Asia Pac. J. Med Toxicol..

[8] Noori N., Boryri T., Teimouri A., Moghadam S.S. (2022). Pediatric poisonings due to chemical substances and related factors. Int. J. High. Risk Behav. Addict..

[9] Tiwari A., Trivedi P., Mishra S., Sachdev M., Panda S., Malini G. (2020). Clinical profile and outcome of poisoning in children admitted to a tertiary care hospital. Asian J. Pedia.

[10] Schwarz E.S., Kopec K.T., Wiegand T.J., Wax P.M., Brent J. (2017). Should we be using the poisoning severity score?. J. Med. Toxicol..

[11] Gossel T.A. (2018).

[12] Pianca T.G., Sordi A.O., Hartmann T.C., von Diemen L. (2017). Identification and initial management of intoxication by alcohol and other drugs in the pediatric emergency room. J. De. Pediatr..

[13] Hahn A. (2020). In Information resources in toxicology.

[14] Çalışkan F., Akça G., Çalışkan B., Akça Ü. (2021). Clinical use of the poisoning severity score in acute pediatric poisoning. J. Exp. Clin. Med..

[15] Barile F.A. (2019). Clinical Toxicology: Principles and Mechanisms.

[16] Lovegrove M.C., Weidle N.J., Budnitz D.S. (2015). Trends in emergency department visits for unsupervised pediatric medication exposures, 2004-2013. Pediatrics.

[17] Lee J., Fan N.C., Yao T.C., Hsia S.H., Lee E.P., Huang J.L., Wu H.P. (2019). Clinical spectrum of acute poisoning in children admitted to the pediatric emergency department. Pediatr. Neonatol..

[18] Zhang Y., Yu B., Wang N., Li T. (2018). Acute poisoning in Shenyang, China: a retrospective and descriptive study from 2012 to 2016. BMJ Open.

[19] Lashin H.I., Sharif A.F. (2023). Evaluation of various scoring systems as predictors of the need for intensive care unit admission and other adverse outcomes among patients with acute clozapine poisoning. Toxicol. Res..

[20] Abdelhamid W. (2021). Evaluation of the severity of poisoning exposures among patients presented to Poison Control Center, Ain Shams University Hospitals, Egypt, in 2019. Ain Shams J. Forensic Med. Clin. Toxicol..

[21] Kandeel F., El-Farouny R. (2017). Study of acute poisoning cases in children admitted to menoufia poison control center (MPCC) during the year (2016). *Ain Shams*. J. Forensic Med. Clin. Toxicol..

[22] Moon J., Chun B., Cho Y., Lee S., Jung E. (2021). Characteristics of emergency department presentations of pediatric poisoning between 2011 and 2016: a retrospective observational study in South Korea. Pediatr. Emerg. care.

[23] Alazab R.M. (2012). Determinants of acute poisoning among children (1-60) months Old at a Poisoning Unit of a University Hospital, Egypt, are employed mothers a risk factor? Retrospective cohort study. J. American Sci..

[24] Disfani F.H., Kamandi M., Mousavi S.M., Sadrzadeh S.M., Farzaneh R., Doolabi N., Rahmani K. (2019). Risk factors contributing to the incidence and mortality of acute childhood poisoning in emergency department patients in Iran: a hospital-based case-control study. Epidemiol. Health.

[25] Çalışkan G., Çizmeci E.A., Ünlü N., Girgin N.K., Iscimen R., Kahveci F.Ş. (2021). From activated charcoal to selective plasma exchange: a retrospective analysis of mushroom poisoning cases treated in the intensive care unit. Turk. J. Intern. Med..

[26] Tyrrell E.G., Orton E., Sayal K., Baker R., Kendrick D. (2017). Differing patterns in intentional and unintentional poisonings among young people in England, 1998–2014: a population-based cohort study. J. Public Health.

[27] Mintegi S., Azkunaga B., Prego J., Qureshi N., Dalziel S.R., Arana-Arri E., Kuppermann N. (2019). International epidemiological differences in acute poisonings in pediatric emergency departments. Pediatr. Emerg. care.

[28] Franklin R.L., Rodgers G.B. (2008). Unintentional child poisonings treated in United States hospital emergency departments: national estimates of incident cases, population-based poisoning rates, and product involvement. Pediatrics.

[29] Hassan B.A., Siam M.G. (2014). Patterns of acute poisoning in childhood in Zagazig, Egypt: an epidemiological study. Int. Sch. Res. Not..

